# The Effects of Zirconium and Yttrium Addition on the Microstructure and Hardness of AlCuMgMn Alloy when Applying In Situ Heating during the Laser Melting Process

**DOI:** 10.3390/ma16155477

**Published:** 2023-08-04

**Authors:** Asmaa M. Khalil, Andrey V. Pozdniakov, Alexey N. Solonin, Tamer S. Mahmoud, Mohammad Alshah, Ahmed O. Mosleh

**Affiliations:** 1 Mechanical Engineering Department, Faculty of Engineering at Shoubra, Benha University, Cairo 11629, Egypt; tamer.abdelmagid@feng.bu.edu.eg (T.S.M.); ahmed.omar@feng.bu.edu.eg (A.O.M.); 2 Physical Metallurgy of Non-Ferrous Metals, National University of Science and Technology “MISIS”, Leninsky Prospekt, 4, Moscow 119049, Russia; pozdniakov@misis.ru (A.V.P.); solonin@misis.ru (A.N.S.); alshakh@misis.ru (M.A.)

**Keywords:** Al-Cu-Mg, laser melting, microstructural features, eutectic elements, laser-melted zone, heating platform

## Abstract

This paper studies the effect of the laser melting process (LMP) on the microstructure and hardness of a new modified AlCuMgMn alloy with zirconium (Zr) and Yttrium (Y) elements. Homogenized (480 °C/8 h) alloys were laser-surface-treated at room temperature and a heating platform with in situ heating conditions was used in order to control the formed microstructure by decreasing the solidification rate in the laser-melted zone (LMZ). Modifying the AlCuMgMn alloy with 0.4 wt% Zr and 0.6 wt% Y led to a decrease in grain size by 25% with a uniform grain size distribution in the as-cast state due to the formation of Al_3_(Y, Zr). The homogenization dissolved the nonequilibrium intermetallic phases into the (Al) matrix and spheroidized and fragmentized the equilibrium phase’s particles, which led to the solidification of the crack-free LM zone with a nonuniform grain structure. The microstructure in the LMZ was improved by using the in situ heating approach, which decreased the temperature gradient between the BM and the melt pool. Two different microstructures were observed: ultrafine grains at the boundaries of the melted pool due to the extremely high concentration of optimally sized Al_3_(Y, Zr) and fine equiaxed grains at the center of the LMZ. The combination of the presence of ZrY and applying a heating platform during the LMP increased the hardness of the LMZ by 1.14 times more than the hardness of the LMZ of the cast AlCuMgMn alloy.

## 1. Introduction

Aluminium and its alloys are widely used in technological applications, particularly transportation. The aerospace industry favours the 2xxx family of Al alloys in particular due to their strength, damage tolerance, and fatigue resistance, as well as high fracture toughness, which allows for broad use in aircraft applications [1,2,3]. These alloys (particularly AA2024) are also essential in several industrial fields such as automotive, construction, marine, and defence due to their density, malleability, conductivity, recyclability, corrosion resistance, and low cost [4,5]. Additional improvements in the microstructure and mechanical characteristics of these alloys through the use of various new technologies, such as additive manufacturing and high-laser-power application, have the potential to make this alloy available in new research and production areas [6,7,8,9,10,11].

However, selective laser melting (SLM), which is a process that is similar to the welding process, has several substantial limitations when processing high-strength Al-Cu-Mg alloys such as AA2024 [12,13,14,15,16]. As a result of the melt pool’s rapid melting and rapid cooling, cracking happens when large residual thermal stress and temperature gradients appear in SLM specimens [10,17,18,19,20]. Additionally, because the melt pool can drop at a rate close to 108 K/s [18], it has been reported that crack formation and propagation typically take place when temperature gradients and residual stresses are considerable.

The aluminium content in the binary alloy depends on the balance of the alloying elements (Al-Cu, Al-Mg, Al-Cr, and Al-Si) in each composition. Mg (1.8 wt%), Cu (4.9 wt%), and Si (0.5 wt%) weight ratios make AA2024 exceptionally difficult to process. Furthermore, evaporation of the alloying elements (such as Mg, due to its low boiling point) may result in element loss, which brings the ratios of weight closer to the peak points of the relative crack sensitivity curves [21], increasing the relative crack sensitivity of the AA2024 alloy significantly. Al-Cu-Mg (2xxx) systems are basic for long and widely used wrought alloys. Cast alloys based on the Al-Cu system include AA206, and the AA224 casting alloy is the American representative of the Al-Cu-Mg system. The main alloying elements and impurities in Al-Cu alloys are Mn, Mg, Ti, Zr, Cr, Fe, Si, and Ni [22]. The most popular alloys of the Al-Cu-Mg system used in additive technologies are 2xxx series wrought alloys, which are close in composition to casting alloys. For example, using the AA 2024 alloy as an example, it has been shown that after selective laser deposition, the ultimate strength of this alloy is approximately 400 MPa, while in the cast state, the ingot has a strength of 185 Mpa [15]. It has been shown that with an increase in the energy density of more than 340 J/mm^3^, the relative density of products exceeds 99.5%, and the microcracks and defects disappear entirely. The increase in strength is explained by significantly modifying the dispersion of phases, grains, and solid solution hardening during SLM.

It was shown by Zhang et al. [23] that adding a small amount of Zr leads to the formation of an ultrafine-grained structure, a significant reduction in hot cracks, and an increase in ultimate strength to 450 MPa. One of the problems of using Al-Cu-Mg alloy in laser and AM technologies is its high sensitivity to solidification cracking. Hot (solidification) cracks form during solidification when the alloy undergoes a temperature range with very low ductility. At these temperatures, cracking begins when thermal tensile stresses reach the limit of the endurable strain essential to form a crack during solidifying [17]. Rapid solidification and high cooling rates can cause solidification cracking in Al alloys, according to recent research on the laser cracking susceptibility of this material [16,24,25,26]. The second way to control the solidification cracks for this alloy is by preheating the samples before laser processing. Preheating minimizes the possibility of solidification cracking. The lower induced strain rate in preheated samples is what reduces the fracture initiation tendency, not the slower backfilling feeding rate. Crack growth from fusion lines between pulses cannot be stopped by efficient preheating due to the greater local strain and lower liquid flow rates. On the other hand, if cracks from earlier stressed pulses are allowed to propagate, no new cracks will be initiated during subsequent pulses [27].

In most cases, a concentration of about 5% Cu is most effective; this is deduced from a double-phase diagram [28]. During heating for quenching, the phases of crystallization origin should completely go into solid solution. However, this is not always possible due to the presence of some other additives. During crystallization, up to 1% Mn is added, such that it forms a vital component of the composition (Al). Almost the entirety of the manganese is released as Al_20_Cu_3_Mn_2_ dispersoids upon heating for quenching [28]. Magnesium is introduced into American cast alloys, usually in an amount of up to 2%, which leads to the formation of the S (Al_2_CuMg) phase, which precipitates the composition of the ternary eutectic in nonequilibrium crystallization. Subsequent ageing results in metastable alterations that greatly improve the material’s strength. The most popular problem during the laser melting of cast Al-Cu alloys is the initiation and propagation of solidification cracks [18,29]. In this context, the aim of this study is to modify and suit high-strength AlCuMgMn alloy for laser melting and additive manufacturing processes by adding a combination of the refining element zirconium (Zr) and eutectic element yttrium (Y) to the cast alloy, carrying out homogenization annealing prior to laser melting, and applying a heating platform during the laser melting process to decrease the maximum possible defects that form during high laser melting applications.

## 2. Materials and Methods

### 2.1. Alloy Preparation

Table 1 shows the chemical composition of the alloys, which were cast in a laboratory induction furnace (Interselt, Saint-Petersburg, Russia) with a graphite–fireclay crucible (Lugaabrasiv, Luga, Russia). The resulting mixture was poured onto a water-cooling copper mould with dimensions of 100 × 40 × 20 mm. The melt was made using 99.99 wt.% Al, 99.95 wt.% Mg, and master alloys of Al-53.6 wt.% Cu, Al-5 wt.% Zr, Al-9 wt.% Mn, and Al-10 wt.% Y. The melted liquid was heated to 850 °C for casting. The rate of solidification cooling was 15 K/s.

### 2.2. Sample Preparation for Microstructure Investigation

The microstructural examination of the samples was carried out using a Struers LaboPoll5 (Struers APS, Ballerup, Denmark) polishing machine via mechanical grinding on SiC papers (grit sizes of 320, 800, 1200, 2400, and 4000) and final polishing with an OP-S silica-based suspension with abrasion size of 0.04 µm. For the microstructural analysis, scanning electron microscopy (SEM) was performed on a Tescan-VEGA3 LMH (Tescan Brno s.r.o., Kohoutovice, Czech Republic) and light optical microscopy (OM) was performed on a Zeiss Axiovert 200 M (Carl Zeiss, Oberkochen, Germany). An energy-dispersive X-ray spectrometer (EDS) XMAX80 (Oxford Instruments Ltd., Abingdon, UK) was attached to the SEM. The grain structure was investigated using OM in polarised light. The prepolished samples were anodised in Barker’s solution for 60 s at 18 V.

### 2.3. Heat Treatment Process

Differential thermal analysis DSC using a Setaram Labsys (Setaram Instrumentation, Caluire, France) calorimeter was used to estimate the melting points of the alloys. Small samples with diameters of 335 mm were cut from ingots, with an empty crucible serving as a reference. The temporal dependence of the temperature differential between the sample and reference cells was determined experimentally using an S-type thermocouple (platinum–platinum–rhodium). The measuring inaccuracy was one degree Celsius. The experiments were conducted at temperatures ranging from 20 to 1000 °C. The pace of heating and cooling was 5 °C/min. In addition, heat treatment was performed in Nabertherm and SNOL fan furnaces with a temperature precision of 1 °K. For 8 h, the ingots were homogenized at 480 °C.

### 2.4. Laser Melting Process

The laser melting process for the investigated alloys was carried out using a MUL-1-M-200 pulse-periodic laser welding apparatus (OOO Latikom, Moscow, Russia) equipped with a Nd:YAG laser operating with a wavelength of 1064 nm and argon gas as a shielding gas. The samples were cut from ingots with a thickness of 1.5–2 mm, polished and cleaned on a Struers LaboPol-5 machine (København, Denmark) using SiC paper with numbers P800–P1200, and then treated with a 10% aqueous NaOH solution and a 15% aqueous HNO_3_ solution. After cleaning the surface and removing the oxide deposit, the samples were wetted and dried to eliminate moisture. Table 2 lists the laser melting parameters used throughout the experiment. Tracks were used to melt the alloys.

### 2.5. In Situ Heating during Laser Melting

**Figure 1:**
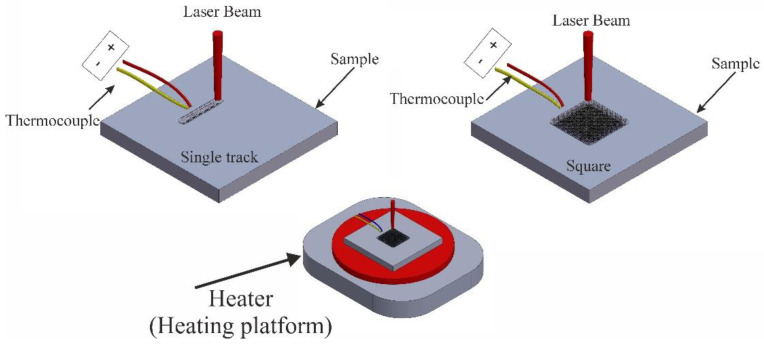
Schematic of applying heating platform during laser melting process.

In this experiment, a heating platform was used under the worked sample during the laser melting process. The temperature range of the heater was from 150 to 450 °C. The heating temperature of the base metal during laser processing was determined using a chromel–alumina thermocouple and using an analogue-to-digital converter at a frequency of 5 kHz which was fixed 5 mm away from the laser melting area, as illustrated in Figure 1. The temperature of the base metal during the laser melting was detected in normal conditions (room temperature) and when using the heating platform.

### 2.6. Hardness and Microhardness

A Vickers hardness testing apparatus with a load of 25 gf and a shutter speed of 15 s was used for measuring the hardness of the laser-melted alloys. The base metal’s hardness values were obtained using an HVD-1000 AP hardness tester machine with HV5, a load of 500 g, and a shutter speed of 10 s.

## 3. Results and Discussion

### 3.1. As-Cast Microstructure

**Figure 2:**
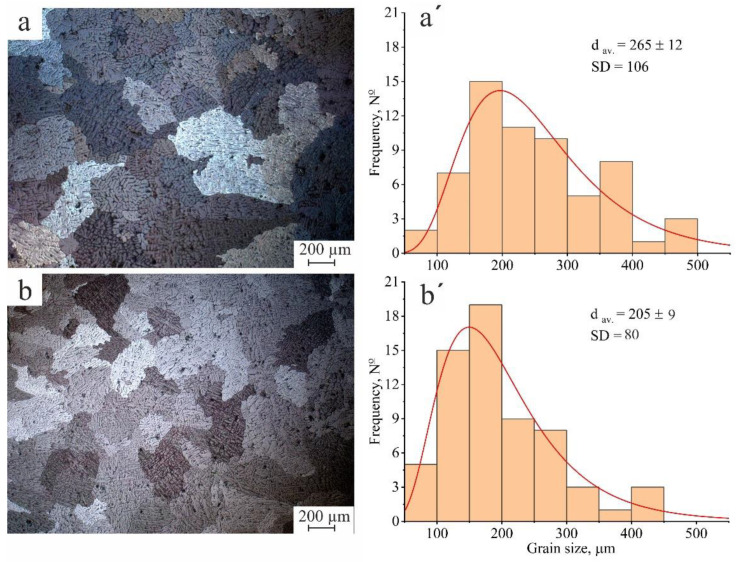
Optical micrographs and grain size–frequency histogram of the (**a**,**a′**) AlCuMgMn and (**b**,**b′**) new modified AlCuMgMnZrY. d_av_, is the average grain size and SD is the standard deviation.

The effect of adding a combination of Zr +Y clearly appeared in the cast state compared with the standard AlCuMgMn alloy, as shown in Figure 2. The standard AlCuMgMn cast alloy has a coarse dendritic random structure with an average grain size of 265 ±12 µm, as shown in (Figure 2a,a′); on the other hand, adding a combination of 0.4%Zr and 0.6%Y slightly refined the dendritic structure, decreased the size and enhanced the distribution of the dendritic grains where the grain size ranged about 205 ± 9 µm (Figure 2b,b′), and decreased the standard division by more than the standard alloy (0.75 times).

**Figure 3:**
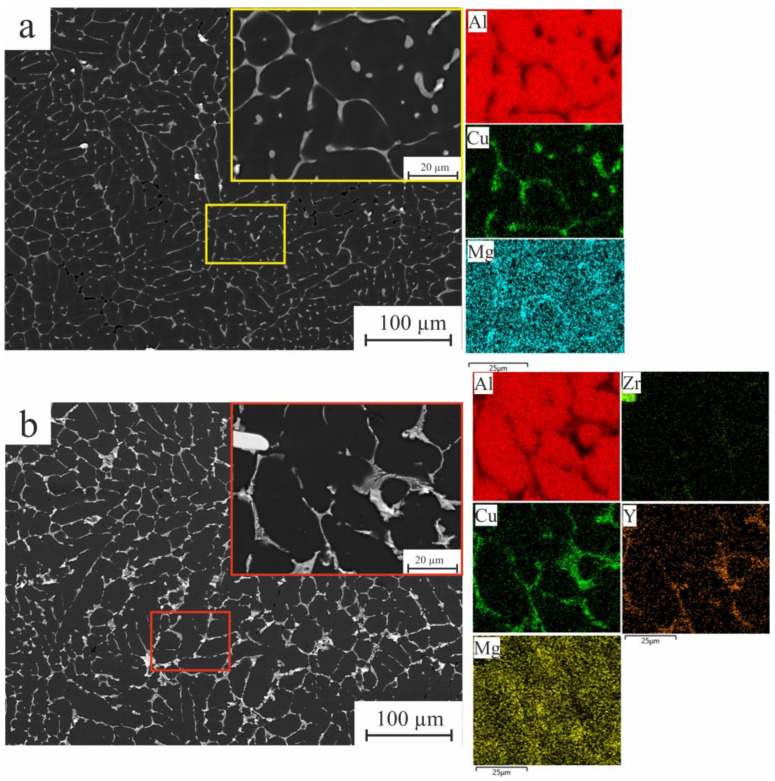
SEM micrograph and map analysis of the alloying elements of the (**a**) AlCuMgMn and (**b**) AlCuMgMn-ZrY alloys.

According to the elemental distribution maps of the alloys as presented in Figure 3a, the microstructure of the as-cast AlCuMgMn standard alloy has primary dendrites of the Al-rich solid solution with small amounts of the elements Mg, Mn, and Cu, in addition to the presence of low-melting-temperature phases θ (Al_2_Cu) in the interdendritic areas. The addition of a combination of Zr and Y resulted in primary Al solid solution dendrites (Al), fine eutectic enriched in Cu, and Y identified in the as-cast microstructure on the grain boundaries with the presence of Al_3_(Zr, Y) phases (see SEM images and maps of the alloying elements at Figure 3b). It can also be noted that the distribution of the ɵ phase became more refined due to the effect of Zr and Y.

### 3.2. Laser Melting of AlCuMgMn Cast Alloy

**Figure 4:**
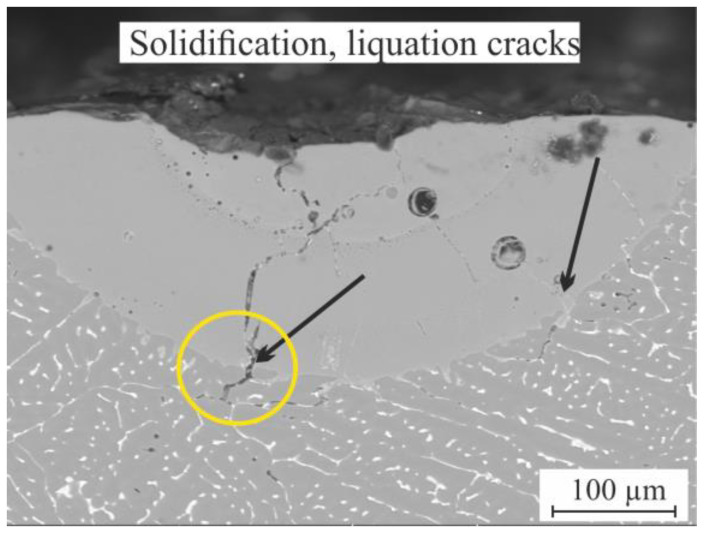
SEM micrograph of the LMZ of the cast AlCuMgMn alloy.

As proved during the previous investigation [18], the presence of low-melting-temperature phases in as-cast alloys (especially the Al_2_Cu phase) is the source of the liquation crack initiation and propagation during the laser melting process. During the laser melting of the cast AlCuMgMn alloy, there were many solidification and liquation cracks (pointed with black arrows) that were initiated from the low-melting-temperature phases at the interdendritic areas at the boundaries of the base metal, as shown in the yellow circle in Figure 4. Thus, homogenization annealing is greatly recommended as a pre-laser-melting process to dissolve the nonequilibrium low-melting-temperature phases and minimize crack initiation during the laser melting process.

### 3.3. Evaluation of the Microstructure during the Homogenization Process

**Figure 5:**
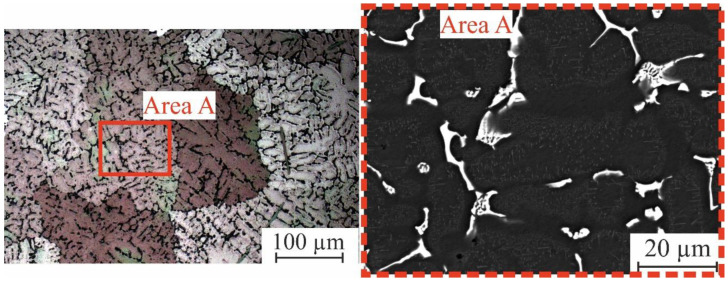
SEM and optical micrographs of the homogenized AlCuMgMnZrY alloy.

Homogenization annealing at 480 °C for 8 h for AlCuMgMnZrY alloy was applied prior to the laser melting process according to the DSC analysis. The suitable homogenization temperature should not exceed 498 °C. This value was determined according to the appearance of the first peak (the peak of the nonequilibrium phases or low-temperature-formed phases) during the heating process for each alloy. The nonequilibrium intermetallic phases were dissolved into the (Al) matrix. Furthermore, as compared to the as-cast state, the particles in the equilibrium phase spheroidized, fragmented, and grew. During solidification, those supersaturated by Y and Zr (Al) should be degraded. As seen in the SEM picture (Figure 5 Area A), after 8 h of homogenization annealing, the tiny precipitates were clearly visible. Figure 5 depicts the microstructure of homogenized AlCuMgZrY, where the grain boundaries grew narrower due to the dissolution of the low-melting-temperature phases during homogenization.

### 3.4. Laser Melting Processing of the Homogenized Alloy

#### Room-Temperature Process

**Figure 6:**
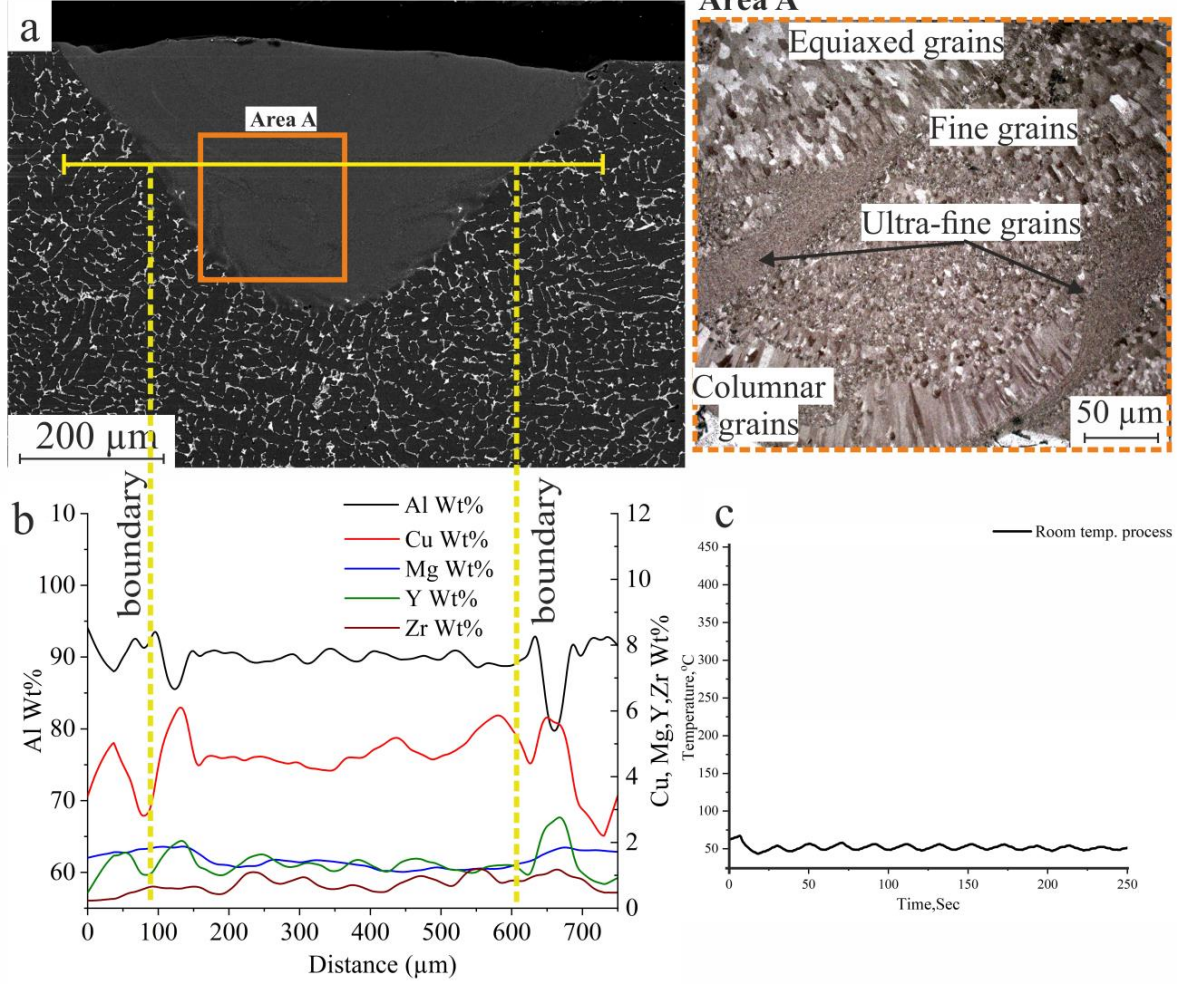
Cross-section micrographs (SEM and optical microscope) of the LMZ: (**a**) single track, (**b**) elemental distribution, and (**c**) the temperature of the BM during the process of AlCuMgMnZrY alloy.

The laser melting process with single tracks was carried out with the previous parameters (listed in Table 2) at room temperature. The temperature during the laser process was around 25 °C; the temperature of the base metal during the laser processing was controlled by a thermocouple that was fixed 1 mm away from the track, as shown in Figure 6c. The microstructure of the laser-melted homogenized AlCuMgMnZrY alloy is shown in Figure 6a. The obtained structure is crack-free, and as shown in Figure 6a which is zoomed in (area A), consists of different types of structures due to rapid solidification and nonequilibrium solidification. The temperature difference between the laser-melted zone (LMZ) and the base metal (BM) caused the nonequilibrium solidification, which reflected on the structure formation, where there is a presence of columnar grains near the boundaries arranged towards the direction of heat release of the BM, in addition to the appearance of small zones of ultrafine grains, and also different sizes of equiaxed grains in the LMZ. The effects of 0.4%Zr and 0.6%Y on the AlCuMgMnZrY alloy as a modifying element and eutectic former, which refined the structure of the LMZ and healed any initiated cracks during the laser melting process, can be observed.

The distribution of elements along the base metal and laser-melted zone can be observed in Figure 6b. There was a slight evaporation of Mg in the LMZ which was around 1.4 wt.% in the base metal and decreased to 1.27 wt.% in the LMZ due to the increasing number of nucleation centres and the refining effect. Zr and Y were nonuniformly distributed along the LMZ (Figure 6b) due to the nonequilibrium and rapid solidification, which significantly affected the hardness and the mechanical properties of the LMZ. The temperature of the BM was controlled during the laser melting process using a thermocouple fixed 5 mm away from the treated track and generated the curve shown in Figure 6c; the recorded temperature of the BM at the investigated structures was around 60 °C.

**Figure 7:**
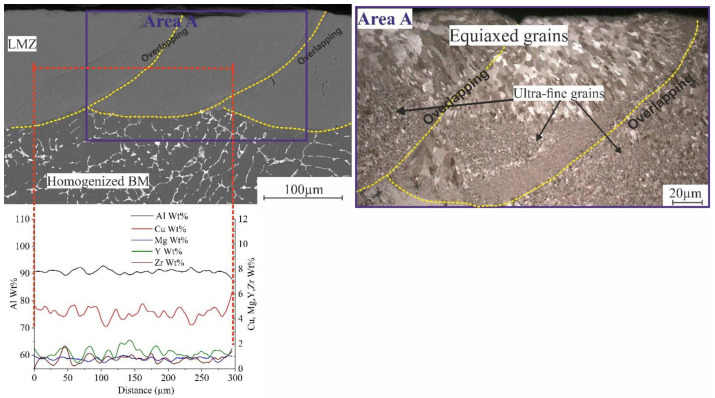
Cross-section of SEM and optical micrographs of the LMZ of multitracks of AlCuMgMnZrY alloy. The yellow dashed lines for overlapping between tracks, the red dashed lines are the boundary of elemental distribution analysis, and the blue is the area A.

The same experiment was carried out by melting multitracks that formed a square with the parameters (listed in Table 2) at room temperature. The obtained structure of the multitracks was a crack-free structure as a single track. The microstructure of the cross-section, as shown zoomed in in Figure 7 (area A), also had different types of structural features due to the temperature difference between the base metal (BM) and the laser-melted zone (LMZ), which led to nonequilibrium rapid solidification. There are ultrafine grains that were formed at the overlapping zones in addition to large zones of equiaxed grains arranged in the direction of laser processing, as shown in the zoomed area (Figure 7, area A). All overlapping is a remelted zone of their previous pulse of the track. So, the presence of the ultrafine grains was due to the temperature difference and the effect of Al_3_(Zr, Y) that enhanced the refining effect, which arranged and concentrated at the boundaries. The average concentration of Mg inside the two sequencer tracks was approximately 0.82 wt.% due to the evaporation of more Mg during the remelting of the overlapping zone for the next track. There was the presence of many peaks of Y and Zr, as shown in the elemental distribution in Figure 7.

### 3.5. Heating Platform Process

**Figure 8:**
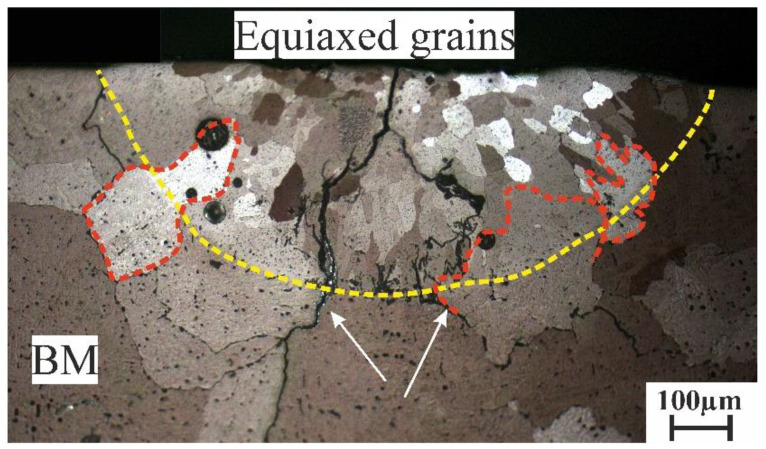
Cross-section micrograph (optical microscope) of the LMZ of a single track using heating platform with a temperature of 410 °C during the process. BM is the base metal unaffected part, the LMZ boundary is indicated by a yellow dashed line and the epitaxial grain growth from BM is indicated by red dashed lines.

**Figure 9:**
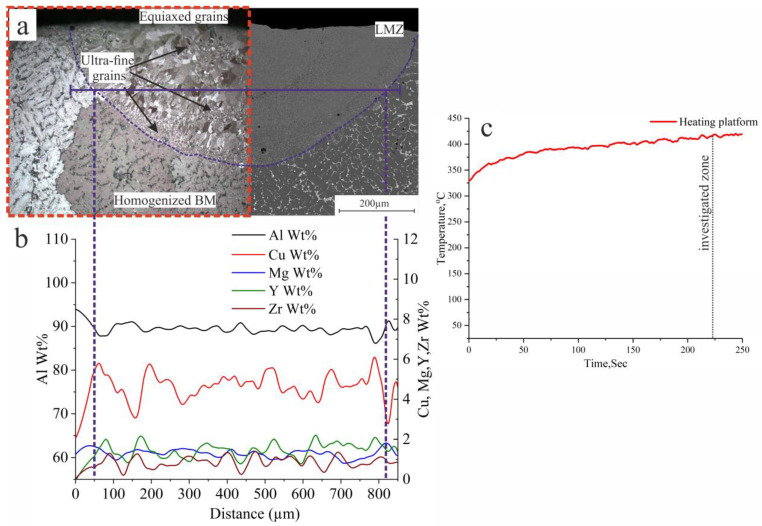
Cross-section micrographs (SEM and optical microscopy) of the LMZ of AlCuMgMnZrY: (**a**) single track, (**b**) elemental distribution, (**c**) the temperature of the BM when using heating platform with a temperature of 410 °C during the process. The optical microscopy is indicated by the red dashed lines. The purple lines are the boundary of the elemental distribution analysis.

The laser melting process was carried out with the parameters listed in Table 2 using a heating platform with a heater with a temperature of about 450 °C during laser melting. During the laser melting process, the BM was heated to change the cooling rate of the melt pool. When the laser began, the temperature of the BM gradually rose. The obtained LMZ during the heating of the reference alloy AlCuMgMn had coarse grains grown epitaxially from the BM and showed the formation of coarse equiaxed grains at the centre of the LMZ, as shown in Figure 8. On the other hand, in Figure 9, the LMZ structures of the cross-section single track during heating had two different zones that had clearly distinguished ultrafine grains at the boundary due to the extremely high concentration of optimally sized Al_3_(Y, Zr) and fine equiaxed grains at the centre of the LMZ; in addition, there were no solidification cracks, and even columnar grains were formed in the LMZ (Figure 9a). The actual temperature of the BM during the laser melting process was about 410 °C, as shown in Figure 9c. Thus, the structure of the first and the end of the track will be obtained at different cooling rates, and different temperature gradients will characterize the crystallization conditions. So, the temperature of the BM will always be lower at the start point and higher at the end. The obtained curve in Figure 9c shows the temperature of the BM during the laser melting of the single track. There was no change in the concentration of Mg evaporation in the LMZ, but there was uniformity in the distribution of Y and Zr, as shown in Figure 9b.

**Figure 10:**
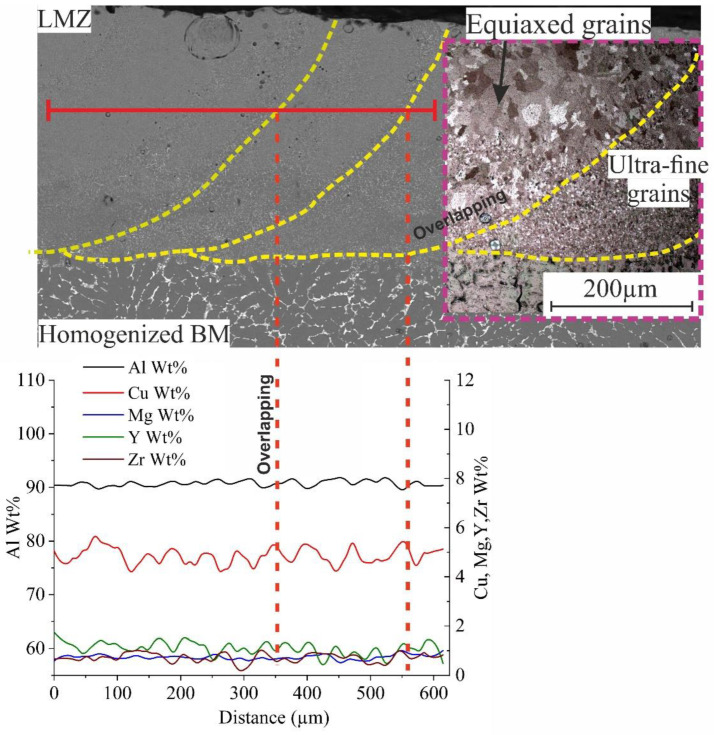
Cross-section micrographs (SEM and optical microscope) of the LMZ of AlCuMgMnZrY with multitracks, and the elemental distribution when using the heating platform with a temperature of 410 °C during the process. The yellow dashed lines for overlapping between tracks, the purple dashed for areas zoomed by optical microscopy, and the red lines are the boundary of the elemental distribution analysis.

In the case of laser-melting several tracks using a heating platform with a temperature of 410 °C, the obtained microstructure had ultrafine grains at the boundaries and overlapping zones, in addition to equiaxed grains at the top of the melting area due to the temperature difference, as shown in Figure 10. The evaporation and oxidation of Mg during the process was higher than in the room-temperature process. The concentration of Mg in the LMZ was about 0.74%.

### 3.6. Hardness of the LMZ

**Figure 11:**
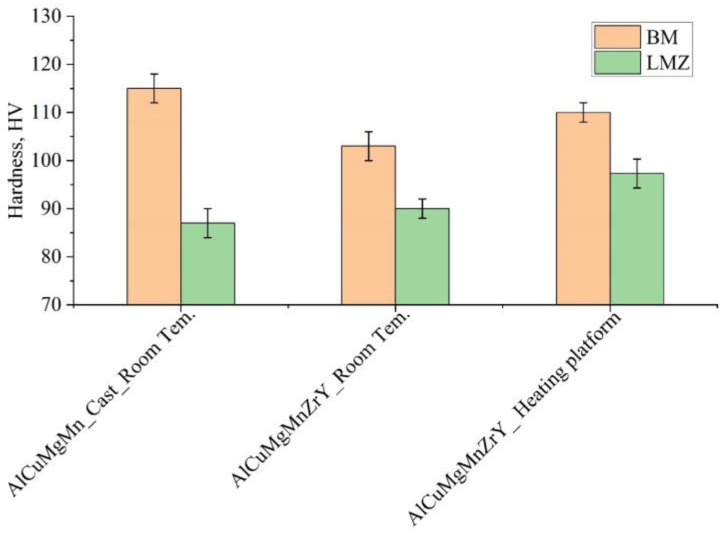
Hardness profiles of the BM and LMZ of the reference alloy, AlCuMgMn, and the modified alloy with ZrY.

Figure 11 shows the average hardness of the base metal (BM) and of the laser-melted zone (LMZ) of the reference alloy (AlCuMgMn) and the modified alloy with Zr and Y. The average hardness of the as-cast reference alloy was 115 ± 4 HV. After the LMP at room temperature, the hardness in the LMZ was significantly decreased to 87 HV; this can be attributed to the evaporation of Mg in the LMZ in addition to the formed defects and cracks in the LMZ. The BM of the homogenized modified alloy exhibited lower hardness, 103 ± 3 HV, due to the dissolving of the formed phases during homogenization. Performing the LMP on the surface of the homogenized modified alloy decreased the hardness of the LMZ more than that of the BM, but it was higher than that of the reference alloy due to the modification of the microstructure and the presence of Al_3_(Zr, Y). Using the heating platform during the LMP helped to increase the hardness of the BM. In this temperature range, the microstructure was modified by forming hardening phases which acted as an aging process. Due to the modification effect, the presence of ultrafine and fine equiaxed grains in the LMZ, and the uniformity of the distribution of the fine grains and the alloying elements along the LMZ, the LMZ exhibited the highest hardness compared to that of the reference alloy and the modified alloys treated at room temperature. It was anticipated that the LMZ’s hardness while using the heating platform would be extremely high, reaching the BM’s hardness, but the high Mg evaporation quantity had a detrimental impact on the hardness.

## 4. Conclusions

The effect of laser melting on the microstructure and hardness values of a reference AlCuMgMn alloy and a modified alloy with 0.4 wt%Zr and 0.6 wt%Y was investigated. By modifying the alloy with 0.4 wt%Zr and 0.6 wt%Y, the grain structure morphology remained dendritic with refined grains (decreased in size by 25%).

The homogenization annealing process dissolved the nonequilibrium phases, homogenized the structure, and uniformly distributed the alloying materials inside the BM, resulting in a homogeneous grain structure in the LMZ and controlling the formation of liquation and solidification fissures. Furthermore, homogenization annealing controlled the production of low-temperature phases and dispersed them evenly, preventing crack formation. After the laser melting process, the microstructure was enhanced by decreasing the temperature difference between the BM and molten pool. By using a heating platform during the LMP to heat up the processed samples, it was achieved. The thermal history of the material and its microstructure is affected by the use of a heating platform during the LMP. Because it is influenced by the melting points and phase transition temperatures, its impact is not the same for all materials. Significant modifications are conceivable depending on the preheating temperature when a phase transformation or precipitation is feasible below that temperature. The heating platform can increase the BM’s temperature, lower the temperature gradients, and even out the temperature distribution. The formation likelihood might, thus, be enhanced to some extent as a result. Due to different temperature gradients, the grain microstructure and performance of the alloy change with the platform temperature.

Using the heating platform around 410 °C during the LMP of homogenized alloy achieved a great improvement, where the solidification cracks completely disappeared, and the hardness of the LMZs after the LMP was significantly improved. The microstructure exhibited ultrafine grains at the overlapping zone and the boundaries. Due to the extremely high concentration of optimally sized Al_3_(Sc, Zr)-based nucleates in these remelt regions, small equiaxed grains were formed preferentially near the top of the track. Thus, the postprocessing heat treatment of LMZs is highly recommended in order to strengthen alloys.

## Figures and Tables

**Table 1:** The chemical composition of the AlCuMgMn alloys.

Alloy	Alloying Elements, wt.%						
	Al	Cu	Mg	Mn	Zr	Y	Others
AlCuMgMn	Bal.	4.9	1.2	1.3	-	-	≤0.1
AlCuMgMn-ZrY	Bal.	5	1.4	1.2	0.4	0.6	≤0.1

**Table 2:** Pulsed laser melting parameters.

Parameter	Unit	Value	Parameter	Unit	Value
Power	V	300	Scanning speed	mm/s	1
Pulse duration	ms	12	Overlap	mm	0.15
Shielding gas	Argon		Frequency	Hz	5
Pulse shape	Ramp-down		Laser diameter	mm	0.2–2.5

## Data Availability

Not applicable.
